# Preoperative albumin-to-fibrinogen ratio as a predictor of postoperative hospital stay in locally advanced esophageal squamous cell carcinoma after neoadjuvant therapy

**DOI:** 10.1038/s41598-025-13603-1

**Published:** 2025-07-29

**Authors:** Hao Chen, Xuan Huang, Yipeng Chen, Weiming Chen, Xin Yan, Chun Chen, Bin Zheng, Haitang Lin, Hanliang Zhang, Chunyu Zhou, Chi Xu, Zhang Yang

**Affiliations:** 1 Department of Thoracic Surgery, Fujian Medical University Union Hospital, Fuzhou, China; 2 Key Laboratory of Cardio-Thoracic Surgery (Fujian Medical University), Fujian Province University, Fuzhou, China; 3 Clinical Research Center for Thoracic Tumors of Fujian Province, Fuzhou, China; 4 National Key Clinical Specialty of Thoracic Surgery, FuZhou, China; 5 Department of Cardiology, Fujian Provincial Hospital, Shengli Clinical Medical College of Fujian Medical University, Fuzhou University Affiliated Provincial Hospital, Fuzhou, China; 6 Department of Second Surgery, Zhangpu County Hospital, Zhangzhou, China; 7 Department of Cardiac Medical Center Nursing, Fujian Medical University Union Hospital, Fuzhou, China; 8 Department of Surgical Nursing, Fujian Medical University Union Hospital, Fuzhou, China

**Keywords:** Albumin-to-fibrinogen ratio, Esophageal squamous cell carcinoma, Neoadjuvant therapy, Length of stay, Preoperative predictors, Cancer, Risk factors

## Abstract

Esophageal squamous cell carcinoma (ESCC) is a major global health issue, with postoperative hospital length of stay (LOS) being a critical factor influencing patient outcomes and healthcare costs. This study evaluates the impact of preoperative albumin-to-fibrinogen ratio (AFR) and albumin-to-D-II aggregates ratio (ADR) on LOS in patients with locally advanced ESCC undergoing neoadjuvant therapy. A retrospective study of 135 patients with locally advanced ESCC who underwent esophagectomy after neoadjuvant therapy (July 2013–November 2020). Demographic, clinical, and preoperative blood data were analyzed. LOS was defined from surgery to discharge. AFR and ADR values were calculated, and ROC curves identified optimal cutoffs. Multivariate Cox proportional hazards models and Kaplan–Meier analysis were used to assess relationships between AFR and LOS. The optimal AFR cutoff was 10.34, demonstrating better predictive accuracy for LOS than ADR. High AFR was associated with significantly shorter LOS. Multivariate analysis revealed high AFR, and cholesterol were linked to shorter stays, while older age and high globulin levels were associated with longer stays. Kaplan–Meier analysis confirmed the relationship. Preoperative AFR is a reliable predictor of LOS in advanced ESCC patients after neoadjuvant therapy, offering potential for improved clinical management and resource allocation.

## Introduction

Esophageal squamous cell carcinoma (ESCC) is the seventh most prevalent malignancy and the sixth leading cause of cancer-related mortality worldwide, posing a significant global health burden^1^. While esophagectomy remains the cornerstone of curative treatment for early-stage ESCC^2^, prolonged postoperative hospitalization (LOS) adversely affects patient prognosis and escalates healthcare costs. LOS serves as a surrogate marker for a patient’s health status during the perioperative period^3^. Reducing LOS can lower healthcare expenditures, minimize the risk of infections and other nosocomial complications, and enhance patient quality of life^4,5^. Consequently, identifying reliable preoperative predictors of LOS is crucial for optimizing perioperative management, curbing healthcare costs, and improving patient outcomes.

Malnutrition, inflammation, and coagulation dysregulation are pivotal in tumorigenesis, progression, and metastasis^6–11^. Patients exhibiting malnutrition, heightened inflammatory responses, or hypercoagulable states often face extended treatment durations, which may prolong LOS^12–14^. Biomarkers such as albumin, fibrinogen, and D-dimer have gained prominence as indicators of nutritional status, inflammation, and coagulation activity. Albumin functions as both a nutritional and prognostic marker, intricately linked to immune and inflammatory responses^15–17^. Hypoalbuminemia is a well-established predictor of poor postoperative outcomes and may contribute to extended LOS^18^. Fibrinogen and D-dimer, coagulation factors associated with tumor cell proliferation and metastasis^19–21^, reflect coagulation and inflammatory activities, thereby influencing postoperative recovery and LOS^22–24^.

The albumin-to-fibrinogen ratio (AFR) and albumin-to-D-dimer ratio (ADR) integrate nutritional, inflammatory, and coagulation parameters, potentially offering superior predictive value for LOS compared to individual biomarkers. AFR and ADR have emerged as promising prognostic indicators in various disorders, including esophageal diseases^25–30^. However, their predictive efficacy for postoperative LOS in patients with locally advanced ESCC undergoing neoadjuvant therapy remains underexplored.

This study aims to elucidate the relationship between preoperative AFR and ADR and postoperative LOS in patients with locally advanced ESCC following neoadjuvant therapy. We hypothesize that lower AFR and ADR levels are associated with prolonged LOS. By identifying patients at elevated risk for extended hospitalization, this research seeks to inform the development of personalized perioperative management strategies, thereby enhancing patient outcomes and optimizing healthcare resource allocation.

## Materials and methods

### Data collection

This retrospective cohort study included adult patients with locally advanced esophageal squamous cell carcinoma (ESCC) who underwent total esophagectomy after neoadjuvant therapy at thoracic surgery department, Fujian Medical University Union Hospital between July 2013 and November 2020. T/N staging was based on preoperative imaging (CT/MRI) following AJCC 8th edition criteria. The study was conducted in accordance with the Declaration of Helsinki and approved by the Ethics Committee of Union Hospital. Written informed consent was obtained from all participants.

### Participant selection

**Fig. 1:**
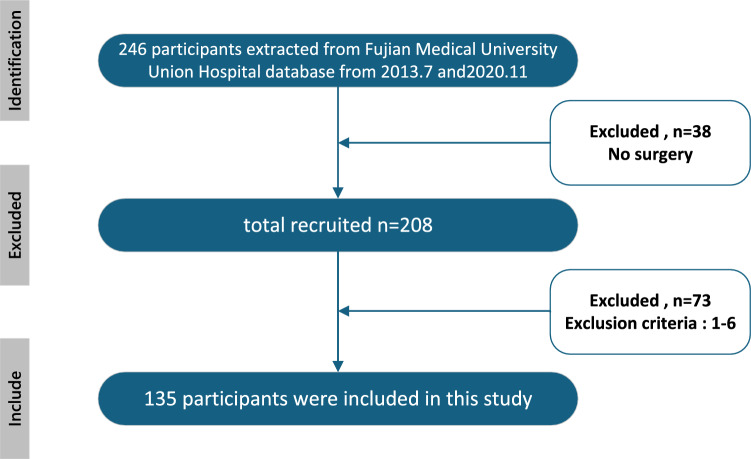
Study flow chart.

Inclusion criteria were: (1) esophageal squamous cell carcinoma confirmed by postoperative histopathology; (2) surgically resectable neoplastic lesions; (3) patients with locally advanced ESCC treated with preoperative chemotherapy( all patients received 2–3 cycles of paclitaxel (175 mg/m^2^) combined with cisplatin (75 mg/m^2^) every 21 days. Surgery was performed 4–6 weeks after completion of chemotherapy); (4) the presence of a single neoplastic lesion. Exclusion criteria were: (1) lung infection, severe chronic obstructive pulmonary disease, pulmonary alveolus, respiratory or cardiac failure, elevated blood glucose, acute or chronic renal insufficiency, significant malnutrition, inflammation, thrombotic disease, or hepatic failure within one month before surgery; (2) multiple primary malignant tumors; (3) admission to the intensive care unit after surgery; (4) missing preoperative laboratory test data; (5) re-admission within three months postoperatively due to poor recovery; or (6) serious postoperative complications were defined as: Clavien-Dindo classification ≥ Grade III. Including: anastomotic leakage, sepsis, respiratory failure, wound dehiscence, massive bleeding, severe infection requiring antibiotics, cardiopulmonary failure, cerebral infarction, and pulmonary infarction. Our study initially included 246 patients diagnosed with locally advanced ESCC after neoadjuvant therapy. During the screening process, 38 patients were deemed unsuitable for surgical intervention due to the advanced stage of their disease. Following the screening, 208 patients remained as potential candidates. After applying the strict inclusion and exclusion criteria, 135 patients who underwent surgery for esophageal squamous cell carcinoma were included in the final analysis (Fig. 1).

### Operational definitions

All minimally invasive procedures were performed by senior thoracic surgeons in our department with extensive experience in minimally invasive esophagectomy (MIE). The operative protocols were standardized across different surgical approaches.

Minimally invasive Ivor-Lewis procedure (abdominal gastric mobilization + right thoracic esophageal dissection + intrathoracic anastomosis):

First, laparoscopic gastric mobilization was performed in the supine position. The greater and lesser omenta were opened, followed by sequential ligation of the short gastric and left gastric arteries. Lymph node stations 1, 2, 3, 4, 7, 8, and 9 were dissected. The distal esophagus was transected using an ENDO GIA™ stapler. The subxiphoid trocar port was extended to 4 cm to exteriorize the stomach for tubular gastric conduit formation. After conduit preparation, pneumoperitoneum was re-established, and the conduit was fixed to the esophageal stump.Next, thoracoscopic esophageal dissection and intrathoracic esophagogastric anastomosis were performed in the left semi-prone position. Single-lung ventilation and artificial pneumothorax (8 mmHg; 1 mmHg = 0.133 kPa) were maintained. The upper mediastinal pleura was incised to dissect recurrent laryngeal nerve lymph nodes. The azygos vein arch was divided, and the thoracic esophagus was fully mobilized with dissection of paraesophageal and subcarinal lymph nodes.After releasing pneumothorax, the 3rd or 4th intercostal trocar port was extended to 4 cm. A purse-string suture or Orvil™ anvil placement method was used to position the stapler anvil, followed by esophageal transection. The gastric conduit was pulled into the thoracic cavity, and the specimen was removed. The conduit was exteriorized through the intercostal incision, and an end-to-side esophagogastric anastomosis was performed using a circular stapler. The gastrotomy was closed with an ENDO GIA™ stapler and reinforced with seromuscular sutures. Finally, the anastomosis was embedded and suspended.

Minimally invasive McKeown procedure (right thoracic esophageal dissection + abdominal gastric mobilization + cervical anastomosis): Thoracoscopic esophageal dissection followed similar steps to the Ivor-Lewis technique but extended cranially to the supraclavicular artery level and caudally to the diaphragmatic hiatus. Unlike the Ivor-Lewis approach, no trocar port extension was required.Laparoscopic gastric mobilization was performed without distal esophageal transection. Cervical anastomosis via a left neck incision: A left sternocleidomastoid incision was made to mobilize and transect the cervical esophagus. The distal stump was closed and tagged with a sterile drainage tube. The subxiphoid trocar port was extended to 4 cm to exteriorize the stomach and esophagus for conduit formation. The conduit apex was attached to the drainage tube and delivered to the neck under laparoscopic guidance (to prevent torsion). A mechanical end-to-side esophagogastric anastomosis was performed, followed by placement of a nasogastric tube and jejunostomy feeding tube.

The decision regarding postoperative discharge was based on the assessment of the supervising physician and the fulfillment of the following criteria: (1) optimal overall health, with adaptation to nutritional fluids and restoration of intestinal function; (2) uneventful recovery from surgical stress, with effective pain control; (3) normal body temperature, absence of positive findings on chest and abdominal examination, and laboratory findings within the normal range or close to it; (4) early discharge from bed activities, leading to improved ability to perform daily activities after discharge; (5) no signs of wound infection, and all drains were removed; (6) postoperative follow-up CT indicating good anastomotic healing. The primary outcome measure was LOS, calculated from the day of surgery to discharge.

The standardized postoperative enteral nutrition protocols at our institution strictly adhere to ESPEN (European Society for Clinical Nutrition and Metabolism) guidelines. Indications for jejunostomy tube feeding include:(1)Oral intake < 50% of basal metabolic requirements within 72 h postoperatively. (2)Preoperative nutritional risk screening (NRS-2002) score ≥ 3. (3)High risk of anastomotic leakage (e.g., preoperative albumin < 30 g/L or intraoperative anastomotic tension).Feeding protocol: Initiation: Nutrison Fibre® (Nutricia) commenced at 20 mL/h within 24 h postoperatively .Escalation: Daily increment of 20 mL/h based on tolerance (gastric residual volume < 200 mL/4 h).Caloric target: 30 kcal/kg/day achieved by postoperative day 5.

### Measured variables

Preoperative demographic and clinical data were collected, including blood samples, age, gender, body mass index (BMI), smoking history, history of respiratory disease, presence of hypertension, presence of diabetes mellitus, tumor localization, degree of tumor differentiation, and TNM stage. Fasting peripheral blood specimens were collected 1–7 days prior to surgery. The routine BMI range was 18.5–23.9 kg/m^2^. The reference intervals for blood biomarkers were: albumin (40–55 g/L), fibrinogen (2.0–4.0 g/L), D-dimer (0–0.5 mg/L), neutrophil count (1.5–7.0 × 10⁹/L), lymphocyte count (0.8–4.0 × 10⁹/L), monocyte count (0.12–0.8 × 10⁹/L), erythrocyte count (3.5–5.5 × 10^12^/L), platelet count (100–300 × 10⁹/L), globulin (20–35 g/L), creatinine (41–73 µmol/L), sodium concentration (135–148 mmol/L), blood pressure (148 mmol/L), and cholesterol (3.40–6.10 mmol/L).

### Data analysis

Continuous variables were expressed as the mean or median, accompanied by standard deviation or interquartile range, depending on the normality of the distribution, and compared using the student’s t-test and Mann–Whitney U-test. Categorical variables were presented as frequencies and percentages, and were compared using the chi-square test. Missing values in clinicopathological variables (respiratory comorbidities, T/N staging, and tumor differentiation grade) were processed using Multiple Imputation by Chained Equations (MICE).The ROC curve is used to calculate the area under the curve (AUC) and to determine the optimal AFR and ADR cut-off points. The DeLong test was employed to compare the performance of the ROC curves for AFR and ADR. LOS was analyzed using the Cox proportional hazards model, with discharge as the outcome event. A hazard ratio (HR) greater than 1 indicated a shorter LOS, while an HR below 1 indicated a longer LOS. Variables with a significance level of < 0.05 in the univariate Cox regression analysis were subsequently included in the multivariate Cox regression analysis. LOS data were described using the Kaplan–Meier method and analyzed using the log-rank test, with discharge as the status variable. The lower Kaplan–Meier curve indicated a shorter LOS, while the upper Kaplan–Meier curve indicated a longer LOS. P-values of < 0.05 were considered statistically significant for all tests. Statistical analyses were performed using SPSS Version 27 software and R version 4.4.2. Time-dependent ROC analysis was performed using the ‘timeROC’ package version 2.0 in R 4.4.2 (R Foundation for Statistical Computing, Vienna, Austria).

## Results

### Optimal cutoff values for AFR

**Fig. 2:**
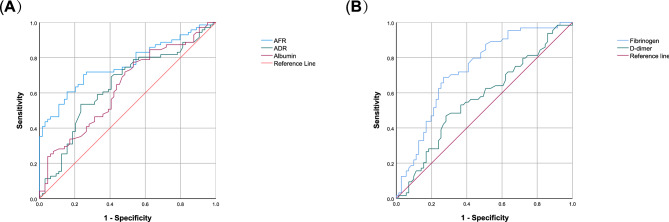
Receiver operating characteristic curves for the ability of preoperative (**A**) albumin, ADR, AFR and (**B**) fibrinogen, D-dimer to predict postoperative hospital LOS for esophageal cancer patients after neoadjuvant therapy. ADR, albumin-to-D-dimer ratio; AFR, albumin-to-fibrinogen ratio; HCC, hepatocellular carcinoma; LOS, length of stay.

ROC curve analysis was performed using albumin, fibrinogen, D-dimer, AFR, and ADR as test variables, with the 10-day median LOS as the state variable. The area under the curve (AUC) values for these variables were 0.631, 0.733, 0.569, 0.761, and 0.641, respectively. AFR demonstrated the highest AUC among all predictors, significantly outperforming ADR (DeLong’s test: p = 0.045). Based on the analysis, an optimal AFR cutoff value of 10.34 was identified. Patients were subsequently categorized into two subgroups: high AFR (> 10.34) and low AFR (≤ 10.34), which were used for further analyses (Fig. 2).

**Fig. 3:**
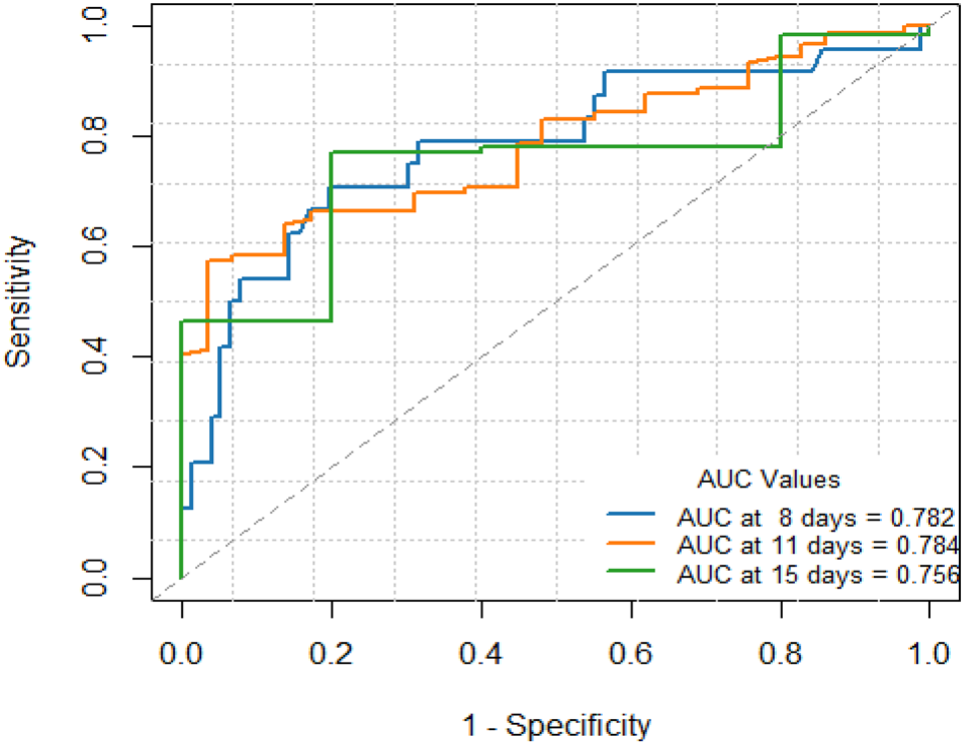
Time-dependent receiver operating characteristic (ROC) curves illustrating the predictive accuracy of preoperative albumin-to-fibrinogen ratio (AFR) for postoperative length of stay in the entire study cohort (n = 135).

In the same time we conducted time-dependent ROC analyses at multiple time points. AUC values remained stable (e.g., 0.782 at 8 days, 0.784 at 11 days), indicating consistent predictive performance of AFR. The slightly lower AUC at 15 days (0.756) likely reflects fewer patients with extended hospitalization (Fig. 3).

### Correlation between clinicopathologic characteristics and AFR

The study cohort included 135 patients, with a median age of 60 years (range:54–65 years) and a male predominance (82.2%). Comparative analyses revealed significant differences between the high-AFR and low-AFR groups. Patients in the low-AFR group were older (median age: 63 vs. 58.5 years, p = 0.029) and exhibited higher neutrophil, monocyte, and platelet counts, as well as elevated fibrinogen levels, compared to the high-AFR group. In contrast, albumin and sodium levels were significantly higher in the high-AFR group (p < 0.001 and p = 0.002, respectively). Detailed clinicopathological characteristics are summarized in Table 1.

**Table 1:** Relationship between the pretreatment AFR and clinicopathological features.

	Total	High-AFR	Low-AFR	p-value
Total(n)	135	66	69	
**Sex(n)**				0.055
Male	111	50	61	
Female	24	16	8	
Age(years, median)	60 (54–65)	58.50 (51–63)	63 (56–65)	0.029
BMI(kg/m^**2**^**,** median)	21.67 (19.98–23.51)	21.396 (19.489–23.009)	21.969 (20.389–23.954)	0.171
**MIE**				NA
Yes	135	66	69	
No	0	0	0	
**Surgical approaches**				0.205
Ivor-Lewis	77	34	43	
McKeown	58	32	26	
Operating time (min)	314 (263–365)	309 (264–354)	318 (263–373)	0.472
**Dissected fields**				0.583
2-field	89	42	47	
3-field	46	24	22	
Harvested mediastinal lymph nodes	18.7 (15.2–22.6)	19.6 (15.9–23.3)	18.2 (14.1–22.3)	0.132
Amount of bleeding (ml)	131 (57–205)	127 (42–212)	136 (65–207)	0.34
Respiratory complications	22	10	12	0.725
Cardiovascular complications	12	5	7	0.6
Other complications	6	3	3	0.956
**Smoking disease**				0.888
Yes	81	40	41	
No	54	26	28	
**Hepatic disease**				0.188
Fatty liver	19	12	7	
Hepatocirrhosis	13	4	9	
Hepatitis B	24	9	15	
No	79	41	38	
**Respiratory diseases**				0.232
Yes	18	6	12	
No	117	60	57	
**High blood pressure**				0.067
Yes	20	6	14	
No	115	60	55	
**Diabetes**				0.975
Yes	2	1	1	
No	133	65	68	
**Tumor localization**				0.808
Upper	13	6	7	
Middle	78	40	38	
Lower	44	20	24	
Erythrocyte count (× 10^**12**^/L, median)	4.17 ± 0.56	4.17 ± 0.54	4.17 ± 0.58	0.927
Platelet counts (× 10^**9**^/L, median)	222.15 ± 62.85	200.7 ± 56.786	242.90 ± 61.860	< 0.001
Albumin (g/L)	41.15(38.375–43.575)	42.6 (40.2–44.7)	39.45 (37.475–42.4)	< 0.001
Globulin (g/L)	29.864 ± 4.62	29.20 ± 4.19	30.505 ± 4.95	0.179
Neutrophil counts (× 10^**9**^/L, median)	3.275 (2.725–4.433)	3.12 (2.65–4.18)	3.615 (2.935–5.0675)	0.041
Lymphocyte count (× 10^**9**^/L, median)	1.744 ± 0.60	1.6985 ± 0.576	1.7876 ± 0.624	0.353
Monocyte count (× 10^**9**^/L, median)	0.465 (0.37–0.60)	0.42 (0.3325–0.515)	0.50 (0.395–0.66)	0.004
CREA (μmol/L, median)	75 (62.75–84)	71.5 (60–84)	75.5 (66–84)	0.305
Na + (mmol/L, median)	140.84 ± 2.457	141.565 ± 2.318	140.14 ± 2.401	0.002
Cholesterol (g/L)	5.2 (4.4250–6.2325)	5.155 (4.415–5.8875)	5.26 (4.4125–6.6525)	0.37
Fibrinogen (g/L)	3.965 (3.385–4.81)	3.41(3–3.8)	4.785(4.145–5.73)	< 0.000
D-dimer (ug/mL)	0.625 (0.367–1.065)	0.59(0.35–0.9075	0.71(0.4175–1.7725)	0.074
**T**				0.209
0	9	3	6	
1	18	13	5	
2	26	14	12	
3	75	33	42	
4	7	3	4	
**N**				0.18
0	62	29	33	
1	46	25	21	
2	23	10	13	
3	4	2	2	
**Discharge destination**				0.964
Home	131	64	67	
Rehabilitation institutions	4	2	2	
Oral intake on the day of discharge (kcal/d)	1017 (829–1168)	1050 (865–1235)	980 (765–1195)	0.107
Duration of jejunal feeding before discharge (day)	9.7 (7.2–12.4)	8.5 (6.4–10.6)	11.3 (7.7–14.9)	< 0.001

AFR, albumin-to-fibrinogen ratio; BMI, body mass index; CREA, creatinine. Data completeness: 99.3% for respiratory comorbidities, 94.1% for T staging, 97.03% for N staging, and 68.9% for tumor differentiation grade.

### Predictors of postoperative LOS

Univariate Cox regression analysis identified several variables associated with LOS, including AFR, age, and globulin levels (all p < 0.05). In multivariate analysis, high AFR (> 10.34) was independently associated with a reduced LOS (HR = 2.213; 95% CI: 1.405–3.017; p < 0.001). Similarly, elevated cholesterol (> 6.1 mmol/L) predicted shorter LOS (HR = 1.642; 95% CI: 1.127–2.538; p = 0.035). Conversely, older age (> 60 years; HR = 0.695; 95% CI: 0.485–0.994; p = 0.047) and high globulin levels (> 35 g/L; HR = 0.358; 95% CI: 0.188–0.679; p = 0.002) were associated with prolonged LOS. These results highlight the predictive value of AFR and its integration with other clinical variables for postoperative outcomes (Table 2).

**Table 2:** Correlations between LOS and AFR and other clinicopathological factors.

	Univariate analysis		Multivariate analysis	
	Hazard ratio (95%CI)	p-value	Hazard ratio (95%CI)	p-value
**Sex**				
male	reference			
Female	1.394 (0.891–2.181)	0.146	1.863 (1.141–3.042)	0.013
**Age**				
≤ 60	reference			
> 60	0.668 (0.474–0.942)	0.022	0.695 (0.485–0.994)	0.047
**BMI**				
≤ 23.9 kg/m^2^	reference			
> 23.9 kg/m^2^	0.826 (0.546–1.249)	0.364		
**Tumor localization**				
Middle + Lower	reference			
Upper	0.712 (0.399–1.271)	0.251	0.639 (0.339–1.204)	0.166
**T**				
T1	reference			
T2-4	1.053 (0.697–1.592)	0.806		
**N**				
No Lymph Node Invasion	reference			
Lymph Node Invasion	1.292 (0.918–1.818)	0.141		
**AFR**				
≤ 10.34	reference			
> 10.34	1.917 (1.351–2.719)	< 0.001	2.213 (1.405–3.017)	< 0.001
**Erythrocyte count**				
≥ 3.5 *10^12/L	reference			
< 3.5 *10^12/L	0.967 (0.521–1.798)	0.917		
**Platelet counts**				
≤ 300*10^9/L	reference			
> 300*10^9/L	1.431 (0.831–2.466)	0.196	1.090 (0.574–2.070)	0.792
**Globulin**				
≤ 35 g/L	reference			
> 35 g/L	0.565 (0.332–0.963)	0.036	0.358 (0.188–0.679)	0.002
**Na + **				
≥ 135 mmol/L	reference			
< 135 mmol/L	1.739(0.426–7.092)	0.441		
**Cholesterol**				
≤ 6.10 mmol/L	reference			
> 6.10 mmol/L	0.782 (0.529–1.157)	0.218	1.642 (1.127–2.538)	0.035
**Neutrophil counts**				
≤ 7.0 × 10*9/L	reference			
> 7.0 × 10*9/L	1.6088 (0.810–3.191)	0.174	1.722 (0.844–3.512)	0.135
**Lymphocyte count**				
≥ 0.8 × 10^9/L	reference			
< 0.8*10^9/L	1.440 (0.726–2.856)	0.297	1.530 (0.741–3.159)	0.250
**Monocyte count**				
≤ 0.8 × 10^9/L	reference			
> 0.8 × 10^9/L	0.569 (0.271–1.194)	0.136	0.645 (0.286–1.453)	0.290

### AFR-based survival analysis Of LOS

**Fig. 4:**
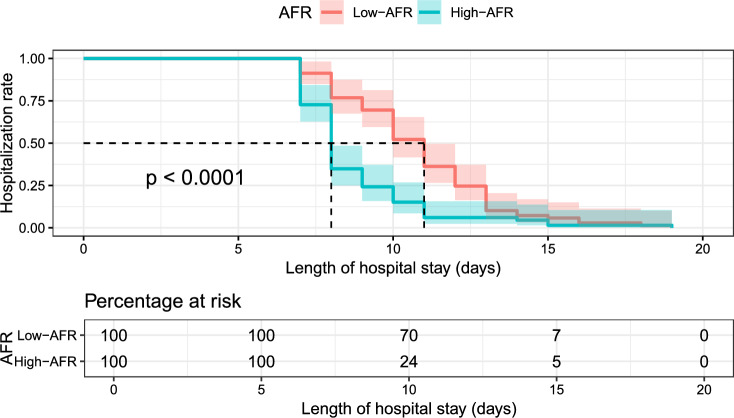
Kaplan–Meier plot of length of hospitalization versus AFR.

Kaplan–Meier survival analysis further confirmed the association between high AFR and reduced LOS. Patients with AFR > 10.34 had a significantly shorter median LOS compared to those with AFR ≤ 10.34 (8 vs. 12 days, p < 0.001). By day 10, fewer than 15% of patients in the high-AFR group remained hospitalized, compared to over 50% in the low-AFR group (log-rank test, p < 0.001) ( Fig. 4).

### Exploratory subgroup analyses

**Fig. 5:**
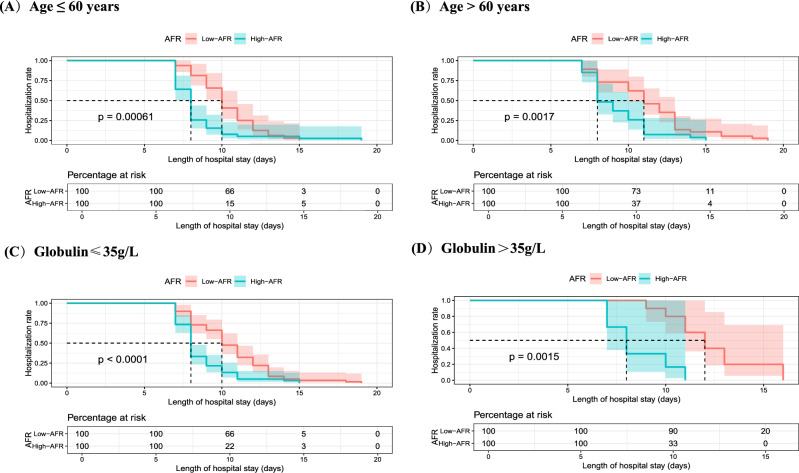
Kaplan–Meier plots of LOS corresponding to the cutoff value of AFR, stratified by (**A**) Age ≤ 60 years, (**B**) Age > 60 years, (**C**) Globulin ≤ 35 g/l, (**D**) Globulin > 35 g/l. AFR, albumin-to-fibrinogen ratio; LOS, length of stay.

Subgroup analyses were conducted to evaluate the predictive value of AFR across different patient subgroups. Regardless of age (≤ 60 or > 60 years) or globulin levels (≤ 35 or > 35 g/L), high AFR consistently predicted shorter LOS. For patients aged ≤ 60 years, high AFR was associated with a median LOS of 8 days compared to 10 days for low AFR (p = 0.00061). Similarly, patients with globulin levels ≤ 35 g/L and high AFR had a significantly shorter LOS (8 vs. 10 days, p < 0.0001). These findings underscore the robustness of AFR as a predictor across diverse clinical contexts (Fig. 5A-5D).

**Fig. 6:**
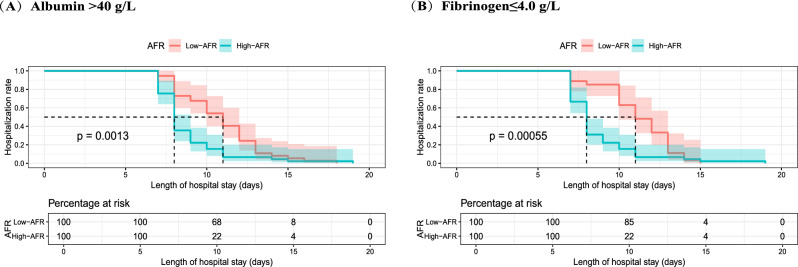
Kaplan–Meier plots of LOS corresponding to the cutoff value of AFR, stratified by (**A**) normal albumin and (**B**) normal fibrinogen levels. AFR, albumin-to-fibrinogen ratio; LOS, length of stay.

To assess AFR’s predictive stability, additional analyses were performed in patients with normal preoperative albumin (> 40 g/L) and fibrinogen (≤ 4.0 g/L) levels. Even within this cohort, high AFR (> 10.34) was associated with a shorter median LOS compared to low AFR (8 vs. 11 days, p = 0.0013 and p = 0.00055, respectively). These results further validate AFR as an independent and reliable predictor of postoperative recovery(Fig. 6A-6B).

**Fig. 7:**
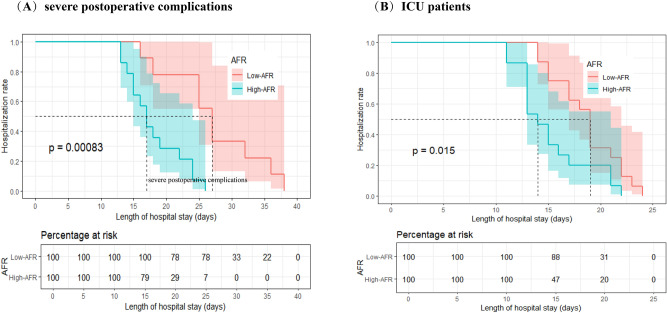
Kaplan–Meier estimates of postoperative LOS according to preoperative AFR status (cut-off 10.34) in two high-risk subgroups. (**A**) Patients who developed severe postoperative complications (Clavien–Dindo ≥ III) but were ultimately discharged (n = 23;p = 0.00083). (**B**) Patients who required unplanned ICU admission after surgery (n = 31;p = 0.015).

Although we excluded patients with severe postoperative complications, we performed subgroup analyses including ICU patients (n = 31, p = 0.015) and the rest patients with severe postoperative complications (n = 23, p = 0.00083). AFR remained a statistically significant predictor of LOS in both groups, confirming its robustness(Fig. 7A-7B).

**Fig. 8:**
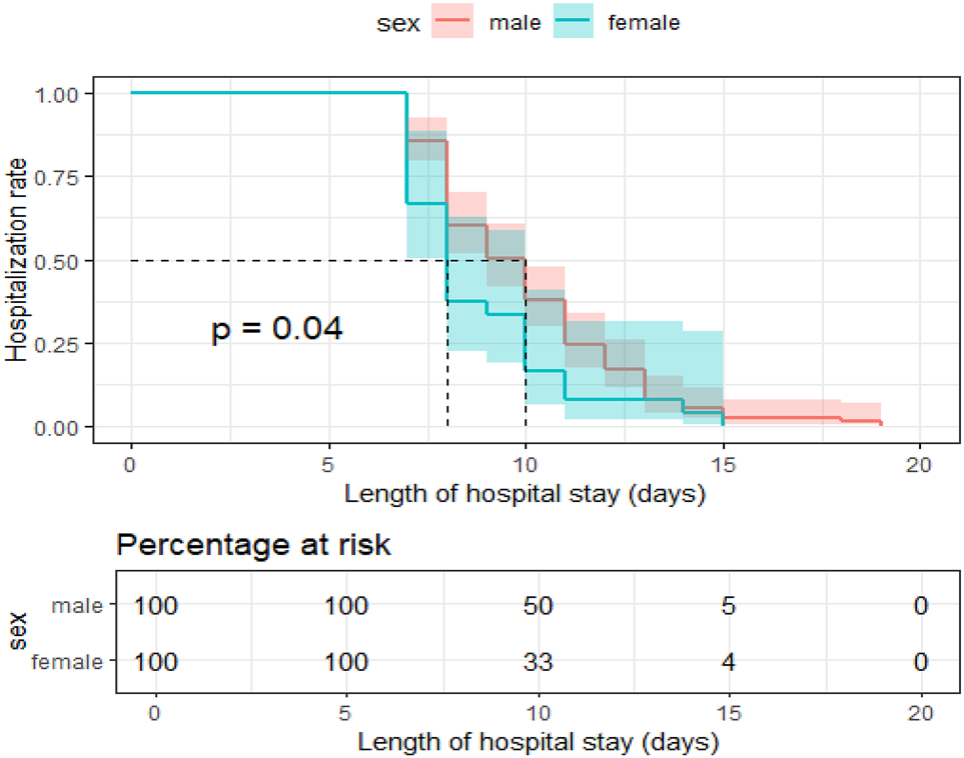
Kaplan–Meier plot of length hospitalization versus AFR values differ based on sex.

There are reports indicating that AFR values differ based on sex, similarly we make a subgroup analysis based on gender. Subgroup analysis revealed that female patients had shorter LOS. Potential explanations include the anti-inflammatory and healing-enhancing effects of estrogen, as well as gender-based differences in immune response and nutritional reserves(Fig. 8).

## Discussion

This study demonstrates the prognostic value of the preoperative albumin-to-fibrinogen ratio (AFR) in predicting postoperative length of stay (LOS) in patients with locally advanced esophageal squamous cell carcinoma (ESCC) following neoadjuvant therapy. To our knowledge, this is among the first investigations to explore AFR as a predictor of LOS in this clinical context. Our findings suggest that AFR is a robust and cost-effective marker for perioperative management, offering valuable insights for improving patient outcomes and optimizing resource allocation.

The results highlight that a higher AFR (> 10.34) is associated with significantly shorter LOS compared to a lower AFR (≤ 10.34). AFR demonstrated superior predictive accuracy over other variables, such as the albumin-to-D-dimer ratio (ADR), as evidenced by its higher AUC (0.761 vs. 0.641, p = 0.045). Elevated AFR likely reflects better preoperative nutritional and inflammatory status, which are critical determinants of postoperative recovery.

Albumin, a vital protein in the human body, plays a multifaceted role in anti-inflammatory, antioxidant, anticoagulant, and antiplatelet aggregation properties, well also preserving colloid osmotic pressure and vascular permeability^31^. Hypoalbuminemia, often indicative of systemic inflammation, impairs the body’s ability to response to surgical stress, correlating with a reduced quality of life and shorter lifespan^32^. It has also been associated with weakened immunity, increased susceptibility to infection, and prolonged hospitalization, despite its potential to promote wound healing and tissue repair after surgery^33,34^. Fibrinogen, a critical component of the hemostatic pathway, play a crucial role in wound healing, tissue injury response, and inflammatory processes^35^. However, elevated fibrinogen levels frequently linked to a hypercoagulable and pro-inflammatory state, which can impede postoperative recovery and prolong hospitalization^36,37^.

AFR reflects a patient’s inflammatory, nutritional, and coagulation status, offering a potential superior prognostic assessment of postoperative recovery compared to albumin or fibrinogen alone. This conclusion is supported by our findings, which show that AFR demonstrated a higher AUC value (0.761) than albumin (0.631) and fibrinogen (0.733). Elevated AFR levels appear to reflect a stronger systemic condition and an enhanced capacity to recover from surgical stress.

Further analysis revealed significant associations between AFR and clinicopathological characteristics. Patients with decreased AFR levels tended to be older and had elevated neutrophil, monocyte, and platelet counts, along with reduced sodium levels. These findings indicate that AFR correlates with multiple clinical parameters and may serve as a predictive marker for both AFR levels and hospitalization duration. There was no significant difference in AFR value in surgery mode, surgical procedure time, blood loss, postoperative complications, lymph node dissection range, etc. Multivariate analysis reinforced these observations, demonstrating that an AFR > 10.34 (HR = 2.213; p < 0.001) and cholesterol > 6.10 mmol/L (HR = 1.642; p = 0.035) positively influenced LOS reduction. In contrast, age > 60 years (HR = 0.695; p = 0.047) and globulin > 35 g/L (HR = 0.358; p = 0.002) were negatively associated with LOS reduction. Integrating AFR with these clinical variables could significantly enhance the accuracy of predicting postoperative hospitalization duration.

Subgroup analyses validated the stability of AFR as a predictor of postoperative LOS. Regardless of age or globulin levels, an AFR > 10.34 consistently predicted a shorter LOS compared to an AFR ≤ 10.34. This finding highlights AFR’s robustness as a predictor that remains unaffected by variations in these parameters. Moreover, AFR was found to be a reliable indicator of postoperative hospitalization duration even in patients with normal preoperative albumin (> 40 g/L) and fibrinogen (≤ 4.0 g/L) levels. Although we initially excluded patients with severe postoperative complications, subgroup analysis showed that AFR was also applicable to this population. From the perspective of gender, women have higher AFR value and shorter hospitalization. This may be due to the anti-inflammatory and healing-enhancing effects of estrogen, as well as gender-based differences in immune response and nutritional reserves^38,39^. These results underscore AFR’s distinctiveness and clinical utility as a predictor of postoperative LOS.

Despite its strengths, this study has limitations. The retrospective design introduces potential biases, such as unmeasured confounders, including operative time and surgeon proficiency, which could influence LOS. While missing data were minimal and appropriately addressed, future prospective studies should prioritize standardized protocols to ensure comprehensive data collection. Additionally, variations in discharge criteria across institutions may limit the generalizability of our findings. Future prospective studies should aim to validate these results in larger, multi-center cohorts and explore AFR’s applicability in predicting other postoperative outcomes, such as complications and mortality. Another limitation is the exclusion of 54 patients (22.3%) with serious postoperative complications. While this approach minimized variability in LOS, it may have restricted the generalizability of the findings to more complex clinical scenarios. Future studies should investigate AFR’s utility in predicting outcomes in such high-risk populations.

AFR represents a simple, cost-effective, and widely accessible marker for risk stratification in ESCC patients undergoing esophagectomy. Its integration into perioperative workflows could aid in tailoring interventions, such as enhanced nutritional support or inflammatory modulation, for high-risk patients. By identifying patients at elevated risk for prolonged hospitalization, AFR may facilitate resource allocation and improve surgical outcomes.

## Conclusion

In conclusion, this study underscores the potential of preoperative AFR as a predictive marker for postoperative LOS in patients with locally advanced ESCC after neoadjuvant therapy. AFR > 10.34 consistently correlates with shorter LOS, making it a valuable tool for optimizing perioperative management and improving patient outcomes. Further research is warranted to expand on these findings and explore AFR’s role in broader clinical settings.

## Data Availability

The datasets used and analysed during the current study available from the corresponding author on reasonable request.
